# The cognitive effects of esketamine: what do we know so far?

**DOI:** 10.1192/j.eurpsy.2022.1879

**Published:** 2022-09-01

**Authors:** M. Barbosa, R. Guedes

**Affiliations:** Centro Hospitalar de Leiria, Department Of Mental Health And Psychiatry, Leiria, Portugal

**Keywords:** esketamine, cognition

## Abstract

**Introduction:**

Esketamine is an S-enantiomer of ketamine approved by the EMA for treatment-resistant depression (TRD). As an NMDA receptor antagonist, its administration results in increase of glutamate release and AMPA receptor activation, supporting both rapid-onset and long-term antidepressant effects. Short-term tolerability seems acceptable but major concerns remain regarding long-term safety, specifically regarding potential neurocognitive toxicity.

**Objectives:**

To clarify the potential short and long-term cognitive beneficial-effects and side-effects of esketamine.

**Methods:**

Research was made using the Medline database, through the Pubmed search engine, using the keywords: “esketamine”, “cognition”. Only randomized-controlled trials were considered.

**Results:**

One study focused on the effects of intranasal esketamine (INE) on cognitive functioning in 24 healthy individuals, who were evaluated pre- and postdose (40 min, 2h, 4h and 6h). The results showed a decline in cognitive performance at 40 min postdose, returning to comparable levels as placebo by 2h postdose. Another study, with a follow-up of 1 year, involving 802 TRD patients, accessed the long-term safety of INE. In patients aged <65 years-old, performance on all cognitive tests remained stable or slightly improved from baseline during long-term treatment. In patients ≥ 65 years-old, the mean performance on all tests improved or remained stable, while the simple and choice reaction time began slowing at week 20.

**Conclusions:**

Esketamine has proven to be a promising new option for the treatment of TRD and available studies have shown promising results regarding patients’ cognitive function. Larger clinical trials are needed to further evaluate its short-term and long-term cognitive effects.

**Disclosure:**

No significant relationships.

